# Farmers’ Perceptions of the Agricultural, Economic, and Health Impacts of Fire Ants in the Brazilian Atlantic Forest

**DOI:** 10.3390/insects17070698

**Published:** 2026-07-04

**Authors:** Victor Hideki Nagatani, Tiago Henrique Nascimento Dativa Vieira, Kelly Carina Braga Bernardo, Samira Daniele Gardziulis Maia Reis, Nathália Sampaio da Silva, Gabriela Procópio Camacho, Otávio Guilherme Morais Silva, Dietrich Gotzek, Maria Santina de Castro Morini

**Affiliations:** 1 Alto Tietê Myrmecology Laboratory, Graduate Program in Biotechnology, Center for Environmental Sciences, University of Mogi das Cruzes, 200 Doctor Cândido Almeida e Souza Avenue–Civic Center, Mogi das Cruzes 08780-911, São Paulo, Brazil; vhnagatani@gmail.com (V.H.N.); tiagohndv@gmail.com (T.H.N.D.V.);; 2 Department of Agribusiness, Mogi das Cruzes Faculty of Technology (FATEC-MC), Vila Nova Mogilar Campus, Mogi das Cruzes 08773-600, São Paulo, Brazil; 3 Laboratory of Systematics, Evolution and Hymenoptera Biology, Museum of Zoology, University of São Paulo (USP), Ipiranga, 481 Nazaré Avenue, São Paulo 04263-000, São Paulo, Brazil; gpcamacho@usp.br (G.P.C.); otavione23@gmail.com (O.G.M.S.); 4 Coordination of Earth Sciences and Ecology, Emílio Goeldi Museum of Pará (MPEG), 1901 Perimetral Avenue, Belém 66077-830, Pará, Brazil; 5 Department of Integrative Taxonomy and Biodiversity of Insects, University of Hohenheim, 70599 Stuttgart, Germany; dietrich.gotzek@uni-hohenheim.de

**Keywords:** rural producers, rural ecosystems, biodiversity conservation, invasive species management

## Abstract

Fire ants are known for their aggressive behavior, omnivorous diet, and construction of mounds on the soil surface. Their dispersal is facilitated by trade and habitat fragmentation, which have led to negative impacts on biodiversity, public health, and agriculture in many countries. In Brazil, information about their impacts is scarce and mostly limited to reports from the North Region. In the Atlantic Forest, a biome where most of Brazil’s population resides, there are no records of impacts associated with fire ants. This study examined farmers’ perceptions of the impacts of fire ants in the Atlantic Forest. A questionnaire was administered to collect information on respondents’ profiles, property characteristics, perceived impacts of fire ants, management practices, and health-related issues. Most respondents reported the regular presence of fire ants on their properties, although the perceived impacts on agricultural productivity were generally low to moderate, and control costs were typically less than $17. Widespread use of pesticides for fire ant control is reported by most farmers. Regarding stings, 85.1% of farmers reported having been stung, but only 0.6% required hospitalization. The most common reaction was itching. This pioneering study revealed that, although fire ants are present on many properties within the Atlantic Forest, the reported economic and health impacts are lower than expected, with most farmers experiencing minimal losses. Overall, the results for our sample suggest that the presence of fire ants does not result in significant economic losses for farmers. Nevertheless, fire ants are not overlooked, as non-conservationist control methods are employed. Such practices may lead to colony fragmentation, increasing their abundance and potentially negatively affecting local biodiversity.

## Simple Summary

This study addresses the lack of information on fire ant impacts in Brazil’s Atlantic Forest. The objective was to evaluate damage based on farmers’ perceptions. A questionnaire collected data on properties, impacts, control, and health issues. Most farmers reported frequent fire ant presence, but impacts on productivity were low to moderate, and control costs were usually below $17. Pesticide use was common. About 85.1% of farmers reported stings; severe cases accounted for 0.6% and required hospitalization; and itching was the main reaction. Although fire ants are widely distributed, this study demonstrates that farmers, for the most part, do not suffer significant economic or health impacts. These findings are valuable because they provide the first data for this biome and support better management strategies, helping guide future research and public policies aimed at balancing agriculture, health, and conservation.

## 1. Introduction

Land-use change associated with globalization has wide-ranging effects on insect communities [1,2,3]. Insects are highly diverse and abundant across terrestrial habitats, including native forests, agricultural systems, and urban areas. Their sensitivity to human-driven disturbances, such as habitat degradation and restoration [4], as well as climate change [5], makes them valuable bioindicators of ecological change [6]. In recent decades, the expansion of global trade has facilitated the spread of several insect species beyond their native ranges [7]. Among them, fire ants, which are generalist and highly adaptable, have expanded their distributions to multiple countries [4,7,8] and, in some cases, to new regions within countries [9]. They can be highly invasive [10,11,12], and the process of fragmenting native vegetation and the consequent edge effect makes environments susceptible to invasion [13]. The presence of agricultural inputs, such as fertilizers and pesticides, also contributes to their proliferation [14,15].

*Solenopsis* has 192 species and 21 subspecies worldwide, most of which are found in the Neotropics, especially in South America [16,17]. The genus is traditionally divided into two informal species groups: thief ants, characterized by their habit of stealing food from other ant colonies, and fire ants, named after the burning sensation, caused by their stings [18]. Fire ants are further divided into four groups: *S. virulens* and *S. tridens*, which are monomorphic, and *S. geminata* and *S. saevissima*, which are polymorphic [19].

Fire ants’ nests form earthen mounds on the ground [20] and can also be built on grasses or pavements [21]. They occupy sandy, silty, and, more often, moist clay soils, which favor the distribution of water, minerals, and nitrogen compounds in the colony [22,23,24]. Their nests alter aeration and water infiltration, changing soil properties [24,25,26]. In addition, they stand out for omnivorous and opportunistic feeding habitats [27] and efficient recruitment during foraging [19,28].

Several impacts associated with fire ants have been described around the world, especially when they occupy new habitats, including the following: (1) environmental—with the displacement of fauna, reduction in local biodiversity, damage to ecosystem services [29]; (2) public health—due to stings and allergic processes, as well as being vectors of pathogens [29]; (3) economic—in the USA and Australia, for example, losses of millions in various sectors of the economy are estimated [30,31]. The expansion of the geographical distribution of fire ants in the world is associated with the globalized transport of goods [7], as well as rising temperatures [32,33].

In Brazil, there are gaps in knowledge about the impacts caused by fire ants [29], and here we emphasize the Atlantic Forest, a biodiverse tropical forest that is located along the Brazilian coast, where 70% of the Brazilian population engages in various economic activities [34]. Records of fire ants, of the *Solenopsis saevissima* species group, in this biome are abundant [9], mainly in anthropized areas [35,36,37,38] and related to open sites such as urban centers [39] and crops [38]. Of particular note is the species *S. saevissima* originates along the Brazilian coast [9], but with records throughout Brazil, including biomes such as the Amazon Forest, the Cerrado, and the Pantanal [40,41,42]; and *S. invicta*, native to the central-western region of Brazil, but also recorded in the south and north of the country [42], and considered invasive in the Atlantic Forest [43], with increasingly frequent records, expanding its distribution, especially in the south and southeast of the Atlantic Forest [9].

In the North of Brazil, the negative impacts of fire ants on agriculture and urban areas have been documented [44], with massive infestations recorded in the municipalities of Envira, Eirunepé, and Novas Airupuanã, in the state of Amazonas [29]. In addition, there are records of infestations in crops, causing damage to plantations in the municipality of Dom Eliseu, in the state of Pará [45], although few records of impacts in the rest of Brazil are known, with most studies being conducted outside the country [46]. In this case, we can highlight the Atlantic Forest as a biome that suffers from frequent anthropogenic processes [34], which favor the occurrence of generalist species such as fire ants [47], widely recorded especially in crops [38], but with little information on impacts, control measures, and medical problems associated with stings. Given these reports, this study seeks to understand how farmers who own properties along the Atlantic Forest perceive the damage and impacts caused by fire ants, with a special focus on *S. invicta* and *S. saevissima*, the most prevalent species in the biome [9,37,48]. Farmers’ knowledge can provide important information that can be used to develop strategies for the control and conservation of the Atlantic Forest, as well as tools for public policies for farmers in relation to the presence of fire ants. One previous study reported predominantly negative perceptions of the red imported fire ant, *S. invicta*, owing to its impacts on agricultural production and human health [46]. However, comparable information is lacking for fire ants in the Brazilian Atlantic Forest. Therefore, this study investigated farmers’ perceptions of the impacts of fire ants on agricultural productivity, economic activities, and human health in this region.

## 2. Methods

### 2.1. Study Area

The research was conducted throughout the Brazilian Atlantic Forest, which was subdivided into three study areas to optimize the online recruitment of farmers from different regions (Study Area 1: Northeast, Study Area 2: Southeast, and Study Area 3: South; Supplementary Material S1). Data collection was conducted sequentially within each study area, enabling a structured search strategy and broader geographic coverage of the Brazilian Atlantic Forest. This approach facilitated the inclusion of farmers from diverse regional settings, enhancing the representativeness of the study sample. The Brazilian Atlantic Forest extends from a latitude −3° to −34°, with altitudes from 0 to 2891 m above sea level, with a wide variety of physiognomies and ecosystems. Its relief includes mountains, plateaus, plains, and depressions [34,49]. The Köppen–Geiger climate classification includes the types Af, Am, Aw, Bsn, Cfa, Cfb, Cwa e Cwb [50].

### 2.2. Data Collection and Questionnaire Design

Data were collected through a questionnaire designed to investigate farmers’ perceptions of fire ants. This descriptive and exploratory study was approved by the Research Ethics Committee (REC; protocol no. 63884522.7.0000.5497). Farmers were contacted in two approaches, but the questionnaire was administered online to all of them to avoid bias during the survey. First, face-to-face surveys were conducted at local farmers’ markets and meetings held by agricultural cooperatives in Southeastern Brazil (State of São Paulo), specifically in the Alto Tietê Region (Supplementary Material S1). This Region was selected because it encompasses part of the São Paulo Green Belt, one of the most important horticultural production areas in Brazil and hosts the institution where this research was conducted. Second, an online survey was distributed to farmers across all Brazilian states within the Atlantic Forest domain. Farmers’ contact information was obtained through agricultural cooperatives, local agricultural offices, and municipal departments of agriculture. Additional contacts were identified through Google Maps (Google LLC, Mountain View, CA, USA) searches using keywords such as agriculture, vegetable production, farmers, agricultural cooperatives, horticulture, orchards, market gardening, and fruit production. The collected information was used to create a database containing the address and available contact details of each farm, including telephone numbers and social media accounts [Facebook, Instagram, and WhatsApp (Meta Platforms, Menlo Park, CA, USA)]. Subsequently, all properties were georeferenced and verified as located within the legal boundaries of the Brazilian Atlantic Forest domain according to the official delimitation established by Brazilian legislation [49].

All farmers (*N* = 955) included in the database were then contacted and informed about the objectives of the research project. To make it easier to participate, an online tutorial has been made available with instructions on how to complete the questionnaire, as well as images, biological information (e.g., descriptions of morphological characteristics and nest shapes), and common names of fire ants. This tutorial was made publicly available through the social media channels of the laboratory where the study was conducted https://www.instagram.com/p/DGd-5iavIp7/ (accessed on 1 April 2026). Subsequently, the questionnaire was distributed through Google Forms (Google LLC, Mountain View, CA, USA) to all farmers who agreed to participate. Following this initial outreach effort, the survey remained available online for an additional three months. During this period, supplementary dissemination efforts were undertaken through collaborations with Brazilian research groups specializing in ant biology and ecology, with the aim of increasing participation and expanding the geographic coverage of respondents.

Thus, potential sources of sampling bias were considered during the study design. Geographic bias was reduced by distributing the questionnaire across all Brazilian states within the Atlantic Forest domain and through multiple recruitment channels, including agricultural cooperatives, municipal agricultural agencies, and research groups. Technological bias was minimized by combining face-to-face and online data collection approaches. To facilitate participation by respondents with different educational backgrounds, explanatory materials containing images, biological information, and common names of fire ants were provided. Nevertheless, because participation was voluntary, self-selection bias cannot be completely excluded, as farmers with previous experiences or greater interest in fire ants may have been more likely to participate.

The questionnaire consisted of 37 questions, including 9 open-ended and 28 closed-ended questions. Following the protocol proposed by [46], the questionnaire was organized into four sections (Supplementary Material S2). Section 1 comprised 15 questions addressing the socioeconomic characteristics of the farmers and their properties (e.g., total area and cultivable area). Section 2 included 9 questions on the impacts of fire ants on rural properties, whereas Section 3 contained 3 questions related to management and control practices adopted by farmers. Section 4 consisted of 10 questions regarding health problems and medical incidents associated with fire ant stings. After data collection was completed, all responses were reviewed and screened for duplicate records prior to analysis.

### 2.3. Data Analysis

A descriptive analysis of sociodemographic variables was conducted, including gender, age, and schooling. A bar chart was created to display the top 20 most frequent words, ordered by descending frequency, with a color gradient indicating word frequency. The chart was produced using the “dplyr” package [51] for data manipulation and the “ggplot2” [52] and “cowplot” [53] packages for visualization and plot arrangement.

To characterize farmers’ perceptions of the economic and health impacts associated with fire ants, survey responses were summarized by calculating absolute and relative frequencies (%) for each response category using the “dplyr” package [51]. The frequency distributions were then presented as horizontal stacked bar charts generated with the ggplot function from the “ggplot2” package [52]. Multi-panel figures were assembled using the *cowplot* package [53], allowing individual graphs to be combined into composite figures identified by panel labels. This descriptive approach enabled the identification of the most frequently reported perceptions and concerns among the interviewed farmers. All analyses, figure assembly, and graphical representations were conducted in the R statistical environment version 4.6.0 (R Core Team, R Foundation for Statistical Computing, Vienna, Austria) [54].

## 3. Results

### 3.1. Characteristics of the Participating Farmers and Their Farms

**Figure 1:**
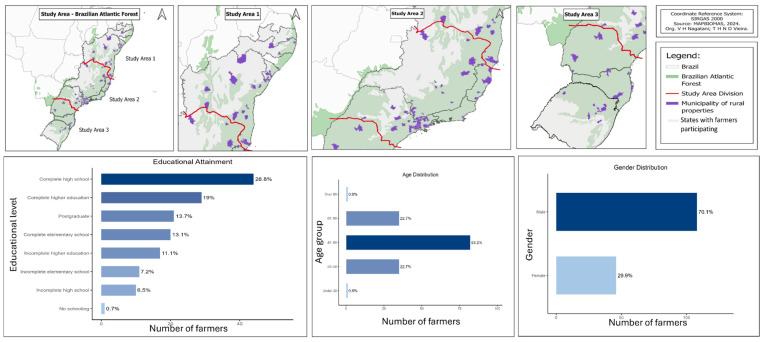
Location of farmers participating in the survey, by state. The graphs show age, education level, and gender. The municipalities in purple are the locations where farmers were interviewed.

A total of 154 farmers in eight Brazilian states (Figure 1) responded to the questionnaire. These farmers have properties ranging from 0.1 to 55 hectares. The cultivable areas range from 30 to 350.00 m^2^. The age of the farmers ranges from 20 to over 80, but the majority (53.2%) are between 40 and 60 years old (Figure 1). The majority (70.1%) of farmers are male (Figure 1) and have completed high school (28.8%) (Figure 1).

**Figure 2:**
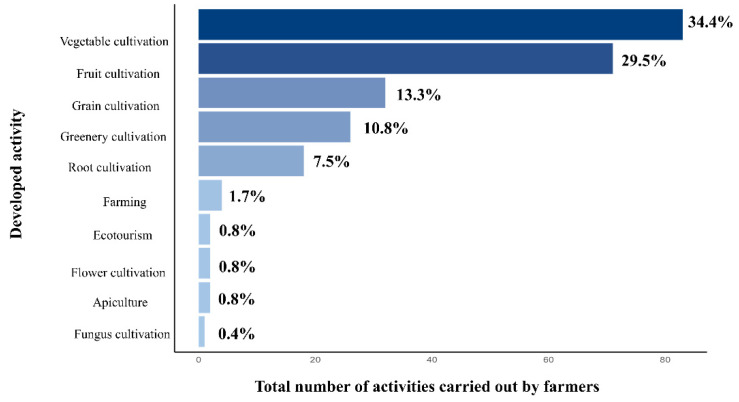
Agricultural activities practiced by farmers with properties in the Brazilian Atlantic Forest.

Vegetables and fruit are the crops most often grown by farmers (Figure 2), who have more than 15 years’ experience (51.9%), or between 5 and 15 years (32.5%), and less than 5 years (15.6%) in these activities.

**Figure 3:**
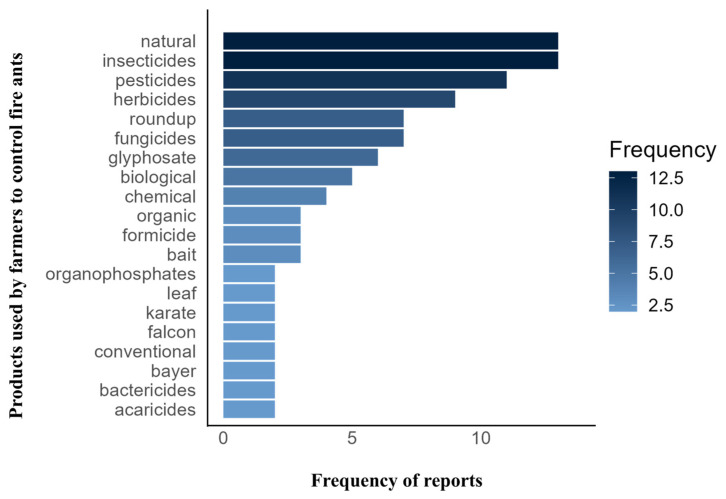
Words used by farmers to describe the types of control used by farmers to combat fire ants.

The majority (66.2%) of farmers carry out conventional practices, using agrochemicals on their properties, generally pointed to the use of agrochemicals, whether specified (e.g., Glyphosate, Roundup, Karate, Falcon) or unspecified (e.g., insecticides, pesticides, herbicides) (Figure 3). While 22.1% practice organic management, they are not registered with the National Organic Production Register. Finally, 11.7% of farmers carry out organic practices and are registered with the National Organic Production Register; most farmers describe natural products without specifying which ones (Figure 3). In this context, the proportion of farmers reporting the use of agrochemicals on their properties indicates that these products predominate in the production system.

The majority (77.2%) of farmers report that the soil on their property is not degraded and shows no change in water conditions, whereas 22.8% report that the soil on their property is degraded and shows changes in water dynamics. With regard to the need to irrigate the soil, 48.7% of farmers describe the need to do it at least once a day, while 14.9% do it once a week and 3.2% once a month. However, 33.1% of farmers report the need to plow the soil once every 3 months, while 6.5% once a week, 4.5% once a month, and 69.5% of farmers do not plow the soil at all.

### 3.2. Diagnosis of the Damage Caused by Fire Ants on Rural Properties

**Figure 4:**
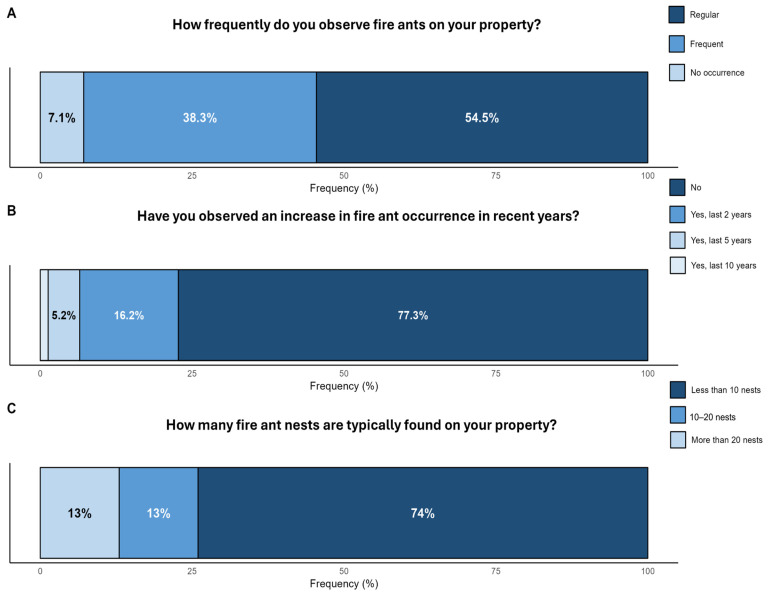
Perceptions of Brazilian farmers with properties in the Atlantic Forest regarding (**A**) the presence of fire ant nests; (**B**) perception of an increase in the number of fire ant nests on properties over time; and (**C**) number of nests on properties.

**Figure 5:**
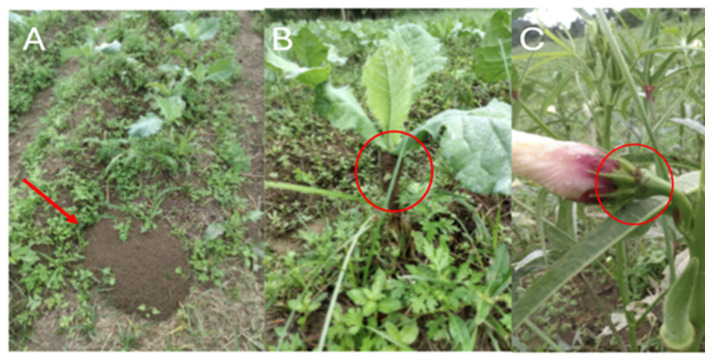
Nest (**A**) and damage caused by *Solenopsis saevissima* in cabbage (**B**) and okra (**C**) crops located in the study area. Arrows and circles indicate nest locations and affected areas on the vegetables, respectively. Source: M.S.C. Morini.

Most farmers describe the presence of fire ants on their properties as moderate (Figure 4A), but they do not perceive an increase in their occurrence in recent years (Figure 4B). The perceived occurrence is less than 10 nests for most landowners (Figure 4C). Most (49.4%) of the nests are located within cultivated areas (Figure 5A), 18.2% are more than 10 m from crops, 16.2% are less than 10 m away, and 16.2% are less than 5 m away.

### 3.3. Farmers’ Responses on the Impacts of Fire Ants on Agricultural Activities

**Figure 6:**
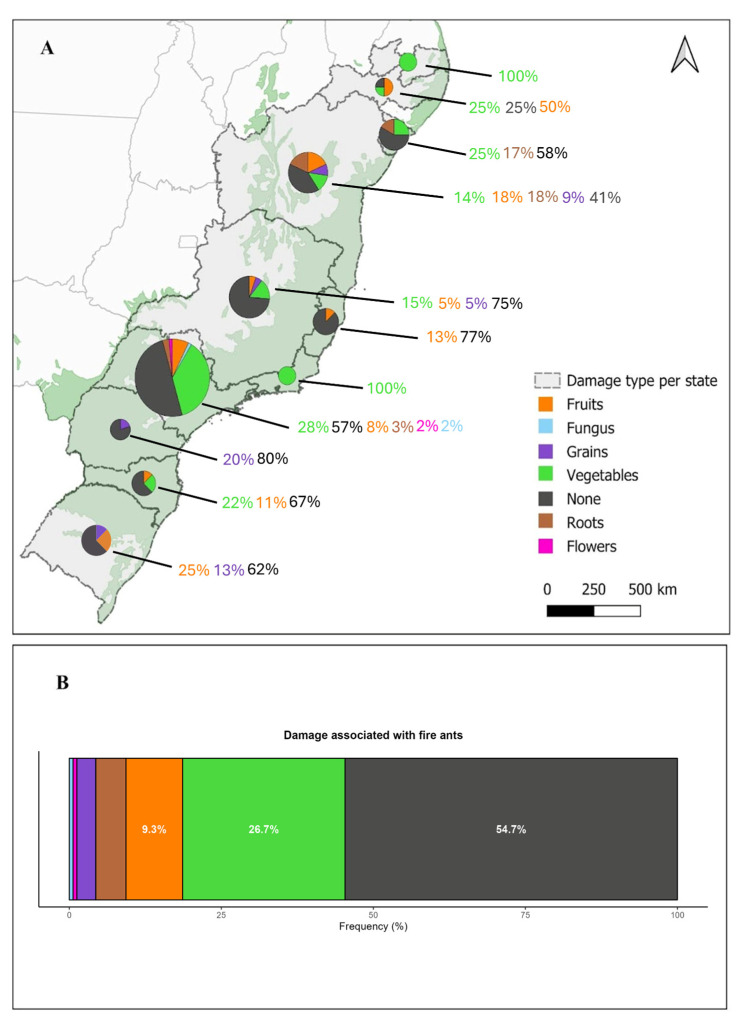
Farmers’ perceptions of crops affected by fire ants, based on farmers with properties in the Atlantic Forest. (**A**) Distribution by state and (**B**) frequency of the main crops identified.

Most farmers (70.8%) report having observed no negative impact of fire ants on agricultural production, while 18.2% report damage of 10% to their crops due to the presence of fire ants on their planted crops (Figure 5B,C); meanwhile, 11% report damage ranging from 11% to 70%. Most farmers report that their crops were not damaged by fire ants (Figure 6A,B).

**Figure 7:**
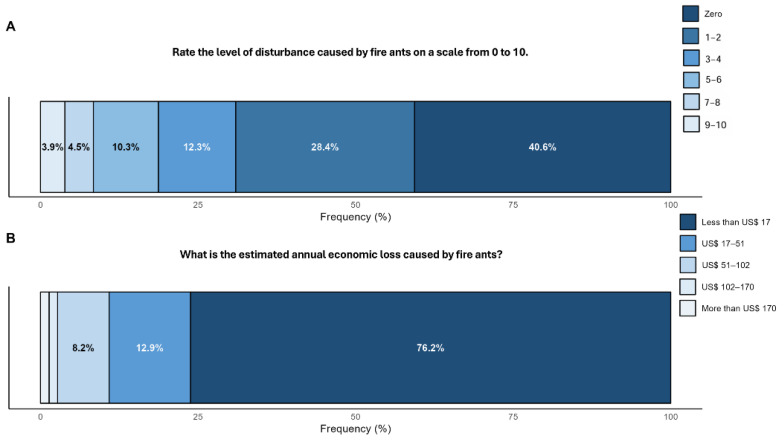
Perceptions of farmers with properties in the Atlantic Forest regarding the impact and damage caused by fire ants to agricultural activities. (**A**) Classification of damage and (**B**) annual expenses.

Based on a rating of the impact of fire ants on agricultural activities on a scale of 0 to 10 (where 0 = no impact and 10 = constant impact), the majority of farmers (40.6%) rated the impact as zero (Figure 7A). Regarding expenses incurred by farmers, most report spending less than US$17 on fire ants per year (Figure 7B).

### 3.4. Diagnosis of Techniques Used by Farmers to Remove Fire Ant Nests on Rural Properties

The majority (60.0%) of the farmers use mechanical control (i.e., manually removing fire ant nests). However, some (21.3%) do not use any control methods. A small percentage (6.5%) use insecticides or alternative methods (12.2%), such as applying hot water with detergent. Among the farmers, 69.9% report repeating the nest removal process once until the structure is eliminated, 19.6% do it twice, 7.8% between three and four times, and 2.6% repeat the procedure more than five times.

### 3.5. Health Damage Caused by Fire Ants on Rural Properties

**Figure 8:**
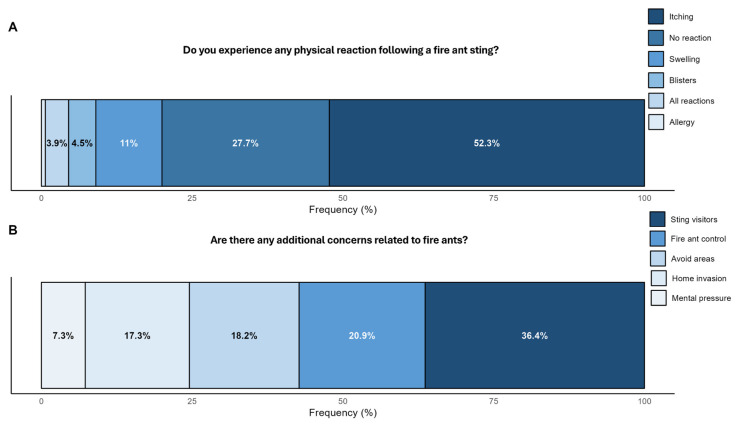
Farmers’ perceptions of the health and psychological impacts of fire ants on their properties in the Atlantic Forest. (**A**) Reactions triggered by fire ant stings and (**B**) concerns reported by farmers with properties in the Brazilian Atlantic Forest.

The majority of farmers (85.1%) report having been stung by fire ants, while 14.9% have never had this experience. Among those who have been stung, 49.4% report being stung at least once a month, 16.9% once a week, and 7.7% daily; the remaining 26.0% report being stung only occasionally. Most farmers reported experiencing itching (Figure 8A) after being stung. Only 0.6% of respondents indicated that they had ever been hospitalized due to the stings. Farmers expressed concerns about fire ants, with most of them worried about visitors being stung (36.4%), followed by ant control (20.9%), avoiding infested areas (18.2%), home invasions (17.3%), and psychological stress (7.3%) (Figure 8B).

Among the group of farmers who have already been stung, the majority (86.7%) report having to spend money on medication at least once a year, while 6.7% spend money every six months and 6.7% spend money every month. Medical expenses are generally less than $17 (65.0%), but some farmers spend between $17 and $102 (25.0%) and, in rare cases, more than $102 (10.0%).

## 4. Discussion

Our work is a pioneering study in Brazil on farmers’ perceptions of fire ants across multiple states of the Brazilian Atlantic Forest, demonstrating the potential of integrating local knowledge into biodiversity monitoring and conservation initiatives [55]. Nevertheless, some limitations associated with the online survey approach should be considered when interpreting the findings. Although online questionnaires facilitate broad geographic coverage, participation may be influenced by factors such as internet access, digital literacy, and educational background [56]. Consequently, certain groups of farmers may have been less likely to participate, potentially leading to the underrepresentation of individuals with more limited access to digital technologies. Furthermore, voluntary participation in online surveys can introduce self-selection bias, as respondents who are more engaged with the topic or more comfortable with digital platforms may be more likely to complete the questionnaire [57]. Therefore, the findings offer an important overview of farmers’ perceptions of fire ants across the Atlantic Forest, although some caution is warranted regarding potential participation biases inherent to online surveys.

The results indicate that, contrary to what is widely documented in other parts of the world, farmers do not perceive fire ants as causing substantial economic losses or risks. This contrasts with the findings of [46], who reported increasing concern regarding the impacts of *S. invicta* in regions where it is considered invasive, particularly because of agricultural damage, risks to human health, and biodiversity loss. In the Brazilian Atlantic Forest, specifically in agricultural systems, fire ant assemblages are predominantly composed of *S. saevissima* [38], a native species that coexists with natural enemies and competing ant species, including other fire ants such as *S. invicta* [47,58]. The predominance of the native species *S. saevissima* in the Atlantic Forest [38] may partially explain the perceptions reported by farmers. Unlike invasive populations of *S. invicta* reported elsewhere [46], native fire ant populations coexist with natural enemies and competing species [47,58], which may reduce their impacts. In addition, the long-term presence of *S. saevissima* in the region may contribute to its perception as a common component of the local environment rather than as a major agricultural threat. However, these hypotheses were not directly tested in the present study and should be evaluated in future research.

This may be a cause for concern due to the homogenization of the Atlantic Forest landscape [59], which leads to the establishment of generalist species and the displacement of specialist species [47], especially in agricultural áreas [14], thereby increasing farmers’ exposure to species such as fire ants and reducing their contact with native and specialist species [59]. These problems are likely to increase in the future, with the rise in human impact, creating challenges for the conservation of the Atlantic Forest [59].

The majority of the farmers are male, a profile similar to that observed by [60,61], and close to that described for Brazilian farmers [62]. The most common age range is between 20 and 60, reflecting the average observed among farmers in Brazil [63]. The majority of the farmers have more than 15 years of experience, a period compatible with that observed in other research with farmers in the Atlantic Forest [61]. In addition, most farmers have completed high school or higher education, which is similar to that found by [61] in the Alto Tietê Region, which is part of the Atlantic Forest. Despite the limitations associated with life in the countryside, such as longer distances to urban centers and fewer transportation options [64], based on [65], farmers in the Atlantic Forest have the intermediate education profile (i.e., completed high school) of modern Brazilian farmers. Sociodemographic characteristics, including educational attainment, can influence local perceptions and attitudes, particularly regarding conservation initiatives within rural communities [66].

Beyond shaping perceptions, educational attainment may also influence farm management practices. Farmers with an intermediate level of education, such as those who have completed high school, tend to adopt conventional management practices, especially when access to technology and machinery is limited [65]. However, farmers with a higher level of education often adopt sustainable or more technologically advanced agricultural practices, typically with more conservation-oriented attitudes [66]. This is, in fact, also reported in the Atlantic Forest, especially in the South and Southeast regions [67]. The continued use of conventional and potentially harmful practices described by farmers (e.g., herbicides, insecticides, and fungicides), in turn, contributes to the ecological imbalance of agroecosystems, favoring opportunistic species such as fire ants. The occurrence and abundance of these ants are frequently associated with disturbed habitats and simplified agricultural systems, where environmental disturbances can reduce biotic interactions and facilitate the establishment of dominant species [14,38].

Most farmers adopt conventional management practices, followed by an organic system, whether registered or not. This profile was observed by [61] among farmers in the Atlantic Forest and is similar to that of farmers in Asian countries, as reported by [46], with occasional differences, such as farm size. The main type of crop is vegetables, which provide ideal habitats for the establishment of *Solenopsis* nests due to the presence of open areas with low canopy cover [35,38], which also favor searching for food resources [6,68]. In addition, the presence of moist clay soils in crops is another relevant characteristic for the presence of fire ants, as they influence the greater distribution of water, minerals, and nitrogen compounds to the colony, particularly for *S. invicta* [22]. In the Atlantic Forest, despite the varied soils (eutrophic and dystrophic soils, on flat and elevations, floodplains, sandbars, and mangroves, as well as frozen and thiomorphic soils, humic soils, and rocky outcrops [69]), in agricultural systems, soil management plays a significant role in the presence of fire ants, as it typically creates conditions conducive to their occurrence [38]. Factors such as soil characteristics (pH, temperature, and density) and constant irrigation are essential for the construction and development of *Solenopsis* nests, as they control temperature and ensure the development of offspring [70,71], which allows *Solenopsis* ants to maintain nests and persist in these environments.

Most farmers described the soil on their properties as undegraded, despite the use of conventional management practices and regular irrigation. However, recurrent disturbance and the use of chemical inputs may favor opportunistic species such as fire ants. Previous studies have shown that *S. invicta* is commonly associated with disturbed and intensively managed habitats [14,38], where anthropogenic activities reduce the diversity of specialized species and facilitate the dominance of generalist ants, often to the detriment of native ant communities [47]. Ref. [38] discusses the maintenance of native vegetation to combat *S. invicta* in anthropized sites and the conservation of *S. saevissima* where they are considered native. The increasingly common presence of generalist species in homogeneous Atlantic Forest habitats, particularly in areas affected by human activity, will be one of the main challenges for conservation, especially in light of climate change, which tends to favor the expansion of fire ants [33,72].

Despite the widespread presence of fire ants, the surveyed farmers generally reported lower perceived impacts on agricultural production than those reported in studies conducted in other regions. In several countries where *S. invicta* is considered an important agricultural pest, studies have documented substantial economic losses and management costs. For example, damage to a variety of crops in the United States led to large-scale federal control programs [21], while annual losses to soybean production have been estimated at between US$31 and US$156 million [30]. Significant expenditures associated with *S. invicta* management have also been reported in Hong Kong [45], Pacific Island countries [73], Australia [31], and China [11]. In Brazil, however, quantitative assessments of economic losses caused by *S. saevissima* in agricultural systems remain scarce, with few published reports, such as impacts on paricá (*Schizolobium amazonicum*) plantations in the northern region of the country [45].

Several factors may contribute to the lower perceived impacts reported by farmers in this study. In regions where fire ants are invasive, natural enemies may be less effective or less abundant, potentially allowing populations to reach higher densities [58]. In contrast, fire ant assemblages in Atlantic Forest crops are often dominated by *S. saevissima* [38], a native species that coexists with local natural enemies and competing species. Although these factors were not directly evaluated in the present study, they may help explain why farmers reported relatively low perceived impacts. Another possible explanation is that the impacts of fire ants may be perceived as less important than those caused by other agricultural pests in Brazil, such as leafcutter ants (*Atta* spp. and *Acromyrmex* spp.), which are widely recognized for causing substantial economic damage [74].

In the European Union (EU), fire ants such as *S. invicta* are classified as pests under Implementing Regulation (EU) 2022/1203. According to the EU, among the problems associated with their presence is the infestation of vegetable crops [75]. The main measures taken to combat them, as described by the EU, include biological control, chemical treatments, sanitizing machinery, and managing the conditions in which nests grow [75]. On the other hand, in Brazil, combat measures are simpler, including manual control and removal or the use of homemade measures (e.g., water and detergent). Thus, our results suggest that the control measures used, regardless of the management applied, seem to be less impactful on the environment, especially given the methods that are discussed in the literature for these farmers in relation to fire ants [76]. Manually removing nests twice by the farmers is usually enough to exterminate them. This was not observed, for example, in Hong Kong, where mechanical removal has also been recorded, but there are extreme cases of infestation. In this case, farmers end up moving their crops to other locations [46].

The methods employed by farmers in the Atlantic Forest are consistent with the main strategies currently used in Brazil to control fire ants [76]. Nevertheless, even though farmers generally reported low perceived impacts, the absence of standardized control procedures deserves attention. Some commonly adopted practices may unintentionally favor colony fragmentation and secondary dispersal [76], particularly when associated with soil disturbance and intensive agrochemical use. As a result, these actions may contribute to the spread of fire ants and the establishment of new infestation sites [14,15]. In addition, the scarcity of specialized technical guidance leads farmers to resort to content from websites and videos available online, which have often not been properly evaluated by experts [76]. Despite this, there are methods that, while often costly, can offer solutions and serve as important tools for the conservation and biological control of fire ants, such as the use of aerial imagery to identify nests [77], genetic monitoring [7], the use of phorids [78], dogs trained to locate nests [79], drier baits [80], or even the use of robot dogs for nest detection [81].

Although the impacts perceived by farmers of fire ants are classified as low, an increase in records of *S. invicta* in Brazil has been noticed, especially in places where these ants are not considered native, such as the Atlantic Forest [9]. Therefore, it is necessary to create standardized processes and provide guidance from public agencies to assist the population [46]. Fast and efficient methods are fundamental for controlling fire ants [79,82,83]. This could be based on cultivation techniques [35,84] or traditional methods [e.g., hot water, formicides, manual removal (see [76])], which are important tools for preventing infestations [35,76]. This may also include the implementation of different techniques combined with monitoring measures, which can be standardized and made accessible to farmers, as proposed in the European Union for fire ant management [75].

Farmers in the Brazilian Atlantic Forest reported being stung by fire ants, with most reactions described as itching and only a small proportion requiring medical attention. This contrasts with reports from some Asian regions (see [46,79,85]), where more severe reactions have been documented. For example, the Department of Animal and Plant Health Inspection in Taiwan reports that common reactions include blisters and swelling and that 7.5% of cases required medical attention because of more serious conditions, such as urticaria, cellulitis, and anaphylactic shock [85]. In comparison, only a small percentage of respondents in our study reported reactions that required medical care.

Although farmers generally reported lower perceived impacts of fire ants on agricultural productivity, health, and management costs than those documented in some international studies, these findings should be interpreted as perceptions of the respondents rather than direct measures of economic damage or health burden. Given the exploratory nature of the study and the limited representativeness of the sample, the results cannot be generalized to all farmers across the Brazilian Atlantic Forest. Nevertheless, they suggest that, within the surveyed group, fire ants were perceived as causing limited severe health impacts, low rates of medical intervention, and relatively low impacts on productivity and management costs. These findings provide an important baseline for understanding how fire ant impacts are perceived by farmers in the Brazilian Atlantic Forest and may support future investigations of the social, economic, and ecological dimensions of human–fire ant interactions.

Indeed, evidence from other regions of Brazil demonstrates that ants can cause substantial impacts under particular ecological and socioeconomic conditions. For example, severe fire ant infestations were reported in the municipalities of Envira in 1993 and Nova Aripuanã in 2008 [29], both in the state of Amazonas, where infestation levels led to the abandonment of properties and required government intervention [86]. Similarly, concerns associated with other ant species have been documented in Brazilian agricultural systems. In cocoa-growing areas, infestations of *Wasmannia auropunctata* (Roger, 1863), popularly known as “pixixica,” have discouraged cultivation in affected areas and generated significant concern among farmers [87]. Infestations of a similar nature have been reported in Hong Kong [46], where concerns about fire ants extend beyond their direct impacts to potential economic losses, particularly those affecting agricultural activities and ecotourism. Likewise, tourists in Pacific island countries have experienced problems associated with fire ant stings, with negative consequences for the tourism sector [73]. Beyond economic effects, some reports highlight concerns related to fire ant management and the psychological burden of working in infested areas. In this regard, research has examined the emotional impacts associated with fire ant occurrence, including possible effects on depression, anxiety, and sleep disorders [79].

Our results indicate that farmers surveyed in the Brazilian Atlantic Forest generally reported low perceived impacts of fire ants on agricultural productivity, economic costs (including crop losses and expenses associated with ant control), and health-related issues resulting from stings. Although infestations have previously been reported in urban and agricultural areas of northern Brazil [29,45], respondents in our study area perceived fire ants as causing relatively limited impacts. This perception contrasts with reports from other regions of the world, where fire ants have been associated with recurrent economic, social, and health concerns, motivating the implementation of management programs [21], regulatory measures [75], and monitoring and inspection efforts [33,88]. Nevertheless, the low level of perceived impacts observed in this study should not diminish the importance of continued monitoring. Climate change and increasing anthropogenic disturbance are recognized as factors that may favor the expansion of fire ant populations [13], particularly in open habitats created by landscape fragmentation, such as agricultural fields and pastures within the Atlantic Forest biome [35,89].

## 5. Conclusions and Implications

The findings presented here provide the first comprehensive exploratory assessment of farmers’ perceptions regarding the impacts of fire ants on productivity, economic activities, and human health in the Brazilian Atlantic Forest, a global biodiversity hotspot and one of the world’s most important tropical forests. Although farmers reported negative effects, these impacts were generally less severe than expected, especially when compared to those documented in regions where fire ants are invasive. Conventional management practices remain the primary method used to control fire ants, and although farmers are frequently stung, these stings are generally perceived as causing only mild symptoms, such as itching. In addition to documenting these perceptions of fire ants, this study establishes an important baseline for future research (e.g., ecological, economic, and public health) in the Atlantic Forest and other Brazilian biomes. The results also provide valuable information to support the development of monitoring programs, management strategies, and public policies aimed at mitigating the impacts of fire ants in Brazil. Such efforts may be particularly important given evidence of fire ant expansion in the Atlantic Forest and the increasing environmental changes associated with habitat fragmentation and land-use modification that may favor their establishment and spread [9].

## Data Availability

The datasets generated by the survey research during and/or analyzed during the current study are available in the Zenodo repository: 10.5281/zenodo.15757954.

## Supplementary Materials

The following supporting information can be downloaded at: https://www.mdpi.com/article/10.3390/insects17070698/s1. Supplementary Material S1—Priority areas for administering the questionnaire to farmers with properties in the Atlantic Forest. The red circle indicates the São Paulo Green Belt and the Upper Tietê Region. Supplementary Material S2—Questionnaire given to farmers with properties in the Brazilian Atlantic Forest. Supplementary Material S3—Details of the project submitted to the Research Ethics Committee (REC/CEP) via the Plataforma Brasil system.
