# Supplementary material for: Farmers’ Perceptions of the Agricultural, Economic, and Health Impacts of Fire Ants in the Brazilian Atlantic Forest
Source: Insects. 2026 Jul 4;17(7):698. doi: 10.3390/insects17070698 (PMC13411350; doi:10.3390/insects17070698)
Supplement: Supplementary file 1 [file insects-17-00698-s001.zip › Supplementary material S2-final.pdf]

Questionnaire

Interviewer:___________________________**Date:** ________________
Municipality: __________________________**Local:**______________________
Georreferecing: Latitude (S):__________________Longitude (W): ___________________
Start time:___________________**Ending time:** _______________________

**PART I – ASSESSMENT OF PARTICIPANTS' CHARACTERISTICS**	
**IA- SOCIOECONOMIC CHARACTERISTICS**	
**Gender**	
( ) Male ( ) Female	
**Age**	
( ) <20 ( ) 20-40 ( ) 40-60 ( ) 60-80 ( ) >80 years	
**Education**	
( ) No schooling ( ) Completed secondary education	
( ) Incomplete primary education ( ) Incomplete higher education	
( ) Completed primary education ( ) Completed higher education	
( ) Incomplete secondary education ( ) Postgraduate studies	
**Activities conducted on the rural property**	
**Do you conduct any agricultural activities on your property?**	
( ) Yes ( ) No	
**Activity performed**	**Production system**
**(1)**	
**(2)**	
**(3)**	
**(4)**	
**(5)**	
**(6)**	
**(7)**	
**How many years have you been engaged in this activity (or these activities)?**	
( ) Less than 5 years ( ) 5 – 15 years ( )more than 15 years	
**Which of the following production systems best describes your property?**	
I am an organic producer in the process of obtaining certification.	
I am an organic producer registered in the National Organic Production Registry.	
I am a conventional producer and use pesticides on my property.	

If you are not an organic farmer, which pesticides and herbicides do you use on your farm/property?
**(1)**
**(2)**
**(3)**
**(4)**
**(5)**
**(6)**
**(7)**
**(8)**
**(9)**
**(10)**

**IB- AREA DIAGNOSIS**	
**Total property area:_________ hectares**	
**What is the total cultivated area?**	
____________________________ m²	
**Soil condition assessment**	
Undegraded soil and/or unaltered soil-water dynamics	( )
Degraded soil and/or altered soil-water dynamics	( )
**Do you irrigate the cultivated area regularly?**	
No	( )
Yes, once a day	( )
Yes, once a week	( )
Yes, once a month	( )
**Do you till or plow the soil regularly?**	
No	( )
Yes, once a week	( )
Yes, once a month	( )
Yes, once every 3 months	( )

APPENDIX B

**PART II – ASSESSMENT OF FIRE ANT IMPACTS ON RURAL PROPERTIES IN THE ATLANTIC FOREST DOMAIN**	
**How frequently do you observe fire ants on your property?**	
Frequent	( )
Regular	( )
No occurrence	( )
**Have you observed an increase in fire ant occurrence in recent years?**	
No	( )

Yes, over the last 2 years	( )
Yes, over the last 5 years	( )
Yes, over the last 10 years	( )
Yes, for more than 10 years	( )
**Rate the level of disturbance caused by fire ants on a scale from 0 to 10.**	
Zero	( )
1-2	( )
3-4	( )
5-6	( )
7-8	( )
9-10	( )
**What is the impact of fire ants on agricultural production?**	
No perceived impacts	( )
Up to 10% reduction in productivity	( )
11–20% reduction in productivity	( )
21–30% reduction in productivity	( )
31-40% reduction in productivity	( )
41-50% reduction in productivity	( )
51-60% reduction in productivity	( )
61-70% reduction in productivity	( )
71-80% reduction in productivity	( )
81-90% reduction in productivity	( )
91-100% reduction in productivity	( )
**Which crops or agricultural products are damaged by fire ants?**	
**(1)**	
**(2)**	
**(3)**	
**(4)**	
**(5)**	
**(6)**	
**(7)**	
**(8)**	
**(9)**	
**(10)**	
**Where are fire ant nests most commonly found within the cultivated area?**	
Within the cultivation area	( )
Less than 5 meters away	( )
Less than 10 meters away	( )
More than 10 meters away	( )
**What is the estimated annual economic loss caused by fire ants?**	
Less than BRL 100	( )
Between BRL100 and BRL 300	( )
Between BRL300 and BRL 600	( )
Between BRL 600 and BRL 1,000	( )
Over BRL 1,000	( )
**How many fire ant nests are typically found on your property?**	
Fewer than 10 nests	( )
10-20 nests	( )
More than 20 nests	( )

APPENDIX C

PART III – ASSESSMENT OF MEASURES ADOPTED BY FARMERS TO CONTROL FIRE ANTS	
**Do you use conventional or alternative methods to control fire ants?**	
I do not use any fire ant control methods	
Yes, I use conventional chemical control methods________________________________	
**How do you control fire ants on your property?**	
Mechanical control (manual nest removal)	( )
Application of hot water to nests	( )
Use of fire to destroy nests	( )
Combination of two or more of the above methods	( )
Application of water mixed with detergent	( )
Other methods:____	( )
**How many times must the control procedure be repeated to achieve satisfactory results?**	
No repetition is required.	( )
The process must be repeated at least twice.	( )
The procedure must be repeated 3–4 times.	( )
The procedure must be repeated more than five times.	( )

APPENDIX D

PART IV – ASSESSMENT OF HEALTH PROBLEMS CAUSED BY FIRE ANTS ON RURAL PROPERTIES	
**Have you ever been stung by fire ants?**	
Yes	( )
No	( )
**How often are you stung by fire ants?**	
Never	( )
Once a month	( )
Once a week	( )
Once a day	( )
More than once a day	( )
**Do you experience any physical reaction following a fire ant sting?**	
No reaction	( )
Swelling	( )
Blisters	( )
Itching	( )
Allergic reaction	( )
Anaphylactic shock	( )
Multiple reactions, primarily swelling, itching, and blisters	( )
**Have you ever been hospitalized due to fire ants stings?**	
Never	( )
Yes, once	( )

Yes, more than once	( )
**Have you incurred medical expenses due to fire ant stings?**	
Yes	( )
No	( )
**If yes, how frequently do these medical expenses occur?**	
Once a year	( )
Once every six months	( )
Once a month	( )
Once a week	( )
**What is the average amount spent on medical expenses related to fire ant stings?**	
Less than BRL 100	( )
Between BRL 100 and BRL 300	( )
Between BRL 300 and BRL 600	( )
More than BRL 600	( )
**Are there any additional concerns related to fire ants?**	
Visitors getting stung	( )
Avoid areas with high concentrations of fire ants.	( )
They feel concerned when working in these areas/mental pressure	( )
Control them	( )
Home invasion	( )
