# Supplementary material for: Farmers’ Perceptions of the Agricultural, Economic, and Health Impacts of Fire Ants in the Brazilian Atlantic Forest
Source: Insects. 2026 Jul 4;17(7):698. doi: 10.3390/insects17070698 (PMC13411350; doi:10.3390/insects17070698)
Supplement: Supplementary file 1 [file insects-17-00698-s001.zip › Supplementary Material S3 pt1.pdf]

DETALHAR PROJETO DE PESQUISA

DADOS DO PROJETO DE PESQUISA

Título Público: Solenopsis invicta Buren, 1972 e Solenopsis saevissima (Smith, F., 1855) no Domínio Atlântico: status de ocorrência e conhecimento de seus impactos.
Pesquisador Responsável: VICTOR HIDEKI NAGATANI
Contato Público: VICTOR HIDEKI NAGATANI
Condições de saúde ou problemas estudados:
Descritores CID - Gerais:
Descritores CID - Específicos:
Descritores CID - da Intervenção:
Data de Aprovação Ética do CEP/CONEP: 01/11/2022

PLATAFORMA BRASIL COORDENADOR PLATAFORMA BRASIL

DADOS DA INSTITUIÇÃO PROPONENTE

Nome da Instituição: ORGANIZACAO MOGIANA DE EDUCACAO E CULTURA SOCIEDADE SIMPLES LIMITADA
Cidade: MOGI DAS CRUZES

DADOS DO COMITÊ DE ÉTICA EM PESQUISA

Comitê de Ética Responsável: 5497 - Universidade de Mogi das Cruzes - UMC/SP
Endereço: Av. Dr. Cândido Xavier de Almeida Souza, 200, Prédio 2, Sala 21-21 - Centro Cívico
Telefone: (11)4798-7085
E-mail: cep@umc.br

CENTRO(S) PARTICIPANTE(S) DO PROJETO DE PESQUISA

CENTRO(S) COPARTICIPANTE(S) DO PROJETO DE PESQUISA

Voltar

Suporte a sistemas: 136 - opção 8
e-mail: suporte.sistemas@datasus.gov.br
Fale conosco: http://datasus.saude.gov.br/fale-conosco

Chat
