# Supplementary material for: Farmers’ Perceptions of the Agricultural, Economic, and Health Impacts of Fire Ants in the Brazilian Atlantic Forest
Source: Insects. 2026 Jul 4;17(7):698. doi: 10.3390/insects17070698 (PMC13411350; doi:10.3390/insects17070698)
Supplement: Supplementary file 1 [file insects-17-00698-s001.zip › Supplementary Material S3 pt2.pdf]

PARECER CONSUBSTANCIADO DO CEP

DADOS DO PROJETO DE PESQUISA

Título da Pesquisa: Solenopsis invicta Buren, 1972 e Solenopsis saevissima (Smith, F., 1855) no Domínio Atlântico: status de ocorrência e conhecimento de seus impactos

Pesquisador: VICTOR HIDEKI NAGATANI

Área Temática:

Versão: 2

CAAE: 63884522.7.0000.5497

Instituição Proponente: ORGANIZACAO MOGIANA DE EDUCACAO E CULTURA SOCIEDADE SIMPLES

Patrocinador Principal: Financiamento Próprio

DADOS DO PARECER

Número do Parecer: 5.733.337

Apresentação do Projeto:

TODAS AS INFORMAÇÕES DESTE ITEM FORAM EXTRAÍDAS DO PROJETO E/OU DO DOCUMENTO INFORMAÇÕES BÁSICAS DO PROJETO.

Segundo o texto do projeto: "As formigas do gênero Solenopsis apresentam 191 espécies e 22 subspécies distribuídas no mundo, sendo a América do Sul a região com maior riqueza. Nesta região, a identificação das espécies é complexa, devido, principalmente, à morfologia semelhante e coloração variada. No Brasil as espécies especialmente Solenopsis invicta e Solenopsis saevissima são amplamente distribuídas, com muitas sobreposições de ocorrência, o que dificulta a delimitação das áreas de ocorrência natural. Em países asiáticos e nos EUA são registrados diversos impactos no meio ambiente, na saúde pública e na economia devido à S. invicta. No Brasil, pouco é abordado sobre este assunto. A partir desta problemática, o objetivo deste trabalho é avaliar a ocorrência, os danos e informações biológicas e de controle de Solenopsis invicta e Solenopsis saevissima no Domínio Atlântico. Para isso serão visitadas coleções biológicas (p.e., MZUSP, MPEG, UFPR, CPDC), coletas serão feitas em diferentes regiões da Mata Atlântica e; serão aplicados questionários para avaliar os danos ocasionados e obter novas informações biológicas e de controle de Solenopsis".

Neste sentido, a pesquisa apresenta como hipóteses que “Os agricultores com propriedades em

locais de Domínio Atlântico são prejudicados pela ocorrência de espécies de Solenopsis invicta e Solenopsis saevissima, principalmente em relação a depreciação dos seus produtos e problemas saúde pública relacionados a ferroada (CHAN e GUENARD,2018), portanto, esperamos que quanto maior a ocorrência, mais danos serão relatados pelos agricultores".

A pesquisa será do tipo descritiva e exploratória. Os sujeitos da pesquisa serão os agricultores que possuam propriedades agrícolas no Domínio Atlântico, que serão convidados por meio das Cooperativas Agrícolas.Toda a pesquisa será realizada de forma online. Os participantes serão agricultores que tenham propriedades dentro do Domínio Atlântico. O procedimento de coleta de dados será realizado por meio da aplicação de um questionário on-line divido em quatro sessões de acordo com Chan e Guenard (2019). A sessão I englobará questões sobre as características dos proprietários bem como de suas propriedades rurais. A sessão II englobará questões que indiquem os prejuízos ocasionados por formigas lava pés nas propriedades rurais. A sessão III englobará questões que indiquem as medidas de combates realizadas pelos agricultores para combater formigas lava pés. A sessão IV englobará questões que indiquem se há relatos médicos descritos pelos agricultores, que foram desencadeados por ferroadas de formigas-lava-pés.

O número máximo de participantes que irá preencher o questionário será de 300 (trezentos) produtores rurais. Após concluído o prazo de disponibilização da pesquisa, caso não atinja o número máximo de participantes, a pesquisa poderá ser encerrada com um número mínimo de 150 (cento e cinquenta).

Como critérios de inclusão, foram informados: "Todos os proprietários rurais entrevistados que possuam de fato ou de direito, abrangendo os que possuem a possa mansa e pacífica na forma do artigo 1238 do Código Civil, Lei Federal 10.406/2002 e que tenham acima de 18 anos, dentro de propriedades inseridas no Domínio Atlântico.", enquanto os critérios de exclusão são: "Serão excluídos os proprietários rurais mansa e pacífica que morem dentro de áreas com presença de Domínio Atlântico e tenham mais de 18 anos, mas que recusem se responder".

O plano de análise dos dados informados pelo pesquisador prevê que "Será utilizado o teste de Kruskall-Wallis (teste a posteriori de Dunn - para dados não normais) e ANOVA (teste a posteriori de Tukey - para dados normais) com post hoc para comparação em pares. Para comparação das variáveis individuais, será usado o teste t (para dados paramétricos) e U de Mann-Whitney (para

dados não
paramétricos). Todos os testes serão realizados utilizando o software BioEstat versão 5.3”.

Como desfechos primário, o pesquisador informa que “A percepção sobre as formigas de fogo em áreas agrícolas em regiões de Domínio Atlântico”.

O orçamento estimado será de aproximadamente (R$ 2.000,00) dois mil reais, para a condução do projeto e é referente a possíveis gastos de estrutura como gastos de energia elétrica, despesas com provedor de internet e aplicativos, em razão da pesquisa ser realizada em ambiente virtual. Todo o custo será financiado com recursos de auxílio do Fundo de Amparo a Pesquisa do Estado de São Paulo (FAPESP) e por recursos do próprio pesquisador.

O cronograma apresentado prevê início da coleta de dados a partir de 01/01/2023 e o término da pesquisa em 31/05/2025.

Objetivo da Pesquisa:
Em texto informado pelo pesquisador, são os seguintes os objetivos deste protocolo de pesquisa:

Objetivo Primário: avaliar a ocorrência e os danos ocasionados por S. invicta e S. saevissima na região de Domínio Atlântico.

Objetivo Secundário: Descrever os danos ocasionados por Solenopsis spp. de acordo com os agricultores que possuam propriedades no Domínio Atlântico.

Avaliação dos Riscos e Benefícios:
Em texto informado pelo pesquisador, são os seguintes os riscos e benefícios deste protocolo de pesquisa:

Riscos: Os participantes estarão vulneráveis a riscos psicológicos na realização da pesquisa em ambiente virtual. Trata-se de stress e constrangimento por responder o questionário, ou possível desconforto. Os riscos são transitórios, de baixa gravidade e probabilidade de ocorrência variável de indivíduo para indivíduo. Para minimizar esses riscos, constará no convite e será colocado aviso antes do agricultor começar a responder o questionário. Será deixado um aviso bem claro que ele

pode sentir-se a vontade para responder cada pergunta no tempo dele(a), e apenas se ele(a) sentir-se seguro. Será enfatizado no início do questionário disponibilizado no Google Forms, por meio de um texto, que não há obrigatoriedade de responder todas as perguntas do questionário e, caso não se sinta confortável para responder alguma pergunta o participante pode interromper sua participação a qualquer tempo. O participante poderá ainda, entrar em contato nos telefones que constam no Termo de Consentimento livre e esclarecido para que sejam tomadas as medidas necessárias de assistência e acolhimento, sem que isso lhe acarrete qualquer ônus.

Benefícios: Os resultados possibilitarão conhecer os impactos ocasionados por formigas de fogo, pouco descrito, além de trazer informações que ainda são escassas no Brasil e que podem ser importantes para o subsidiar políticas públicas para o controle de insetos considerados pragas.

Comentários e Considerações sobre a Pesquisa:

Trata o presente protocolo de projeto original, que se encontra na segunda versão submetida pelo pesquisador em resposta ao parecer consubstanciado número 5.713.838, que foi emitido por este CEP em 20 de outubro de 2022.

O protocolo apresenta como pesquisador responsável VICTOR HIDEKI NAGATANI, como assistente de pesquisa a Profa. Dra. Maria Santina de Castro Morini. Quem assinou a folha de rosto foi O Pró Reitor Acadêmico da Universidade de Mogi das Cruzes, Prof. Cláudio José Alves de Brito. Trata-se de um projeto de Doutorado apresentado ao Programa de Pós-Graduação da Universidade de Mogi das Cruzes como parte dos requisitos para candidatura ao Curso de Doutorado em Biotecnologia.

Nesta versão o pesquisador corrigiu a pendência/inadequação apontada no parecer anterior.

Considerações sobre os Termos de apresentação obrigatória:

Consideram-se itens de apresentação obrigatória:

1. FOLHA DE ROSTO – o documento formaliza a autorização da instituição a que o pesquisador é vinculado a
realizar a pesquisa.

2. TCLE – O Termo de Consentimento Livre e Esclarecido:
O TCLE menciona os procedimentos a que os participantes serão submetidos, os riscos e benefícios da realização da pesquisa e ressalta a garantia dos direitos dos participantes da pesquisa quanto à informação, privacidade e encaminhamento. Indica que os itens mencionados estão sob responsabilidade do pesquisador responsável em consonância à Resolução 466/12 do Conselho Nacional de Saúde e da Norma Operacional nº 001 de 2013 do CNS. As informações aos participantes da pesquisa sobre o acesso ao pesquisador e o CEP estão devidamente contempladas no TCLE.

3. QUESTIONÁRIO: O procedimento de coleta de dados será realizado por meio da aplicação de um questionário on-line dividido em quatro sessões de acordo com Chan e Guenard (2019). A sessão I englobará questões sobre as características dos proprietários bem como de suas propriedades rurais. A sessão II englobará questões que indiquem os prejuízos ocasionados por formigas lava-pés nas propriedades rurais. A sessão III englobará questões que indiquem as medidas de combates realizadas pelos agricultores para combater formigas lava pés. A sessão IV englobará questões que indiquem se há relatos médicos descritos pelos agricultores, que foram desencadeados por ferroadas de formigas lava-pés.

Conclusões ou Pendências e Lista de Inadequações:
O presente protocolo de pesquisa não apresenta pendências e/ou inadequações, estando em consonância com as resoluções e normativas vigentes para a condução de pesquisas envolvendo seres humanos.

Considerações Finais a critério do CEP:
O Comitê de Ética em Pesquisa - CEP, de acordo com suas atribuições definidas na Resolução CNS 466/12, solicita ao pesquisador responsável que encaminhe o relatório final ou cópia da publicação do artigo ou resumo referentes a este projeto, através de NOTIFICAÇÃO, quando da conclusão do projeto.

Havendo qualquer evento adverso ou qualquer mudança no projeto que envolva direta ou indiretamente o participante da pesquisa, deve o pesquisador entrar em contato imediatamente com o sistema CEP/CONEP.

Mudanças que não descaracterizam o projeto aprovado como ampliação de amostra ou vigência do

Endereço: Av. Dr. Cândido Xavier de Almeida Souza, 200, Prédio 2, Sala 21-21 - Centro Cívico
Bairro: Prédio II, sala 2121(UMC) **CEP:** 08.780-911
UF: SP **Município:** MOGI DAS CRUZES
Telefone: (11)4798-7085 **Fax:** (11)4798-7085 **E-mail:** cep@umc.br

projeto, dentre outras, podem ser submetidas através de EMENDA para análise e aprovação por este CEP, antes do término da vigência do projeto.

Quanto ao direito à assistência, segundo o item V.6 da resolução 466/12, "o pesquisador, o patrocinador e as instituições e/ou organizações envolvidas nas diferentes fases da pesquisa devem proporcionar assistência imediata, nos termos do item II.3, bem como responsabilizarem-se pela assistência integral aos participantes da pesquisa no que se refere às complicações e danos decorrentes da pesquisa."

Em relação à proteção dos dados, o pesquisador deve respeitar também na sua pesquisa os termos da lei n. 13.709/18 (LGPD), responsabilizando-se por eventual vazamento ou uso indevido dos dados coletados dos participantes

Este parecer foi elaborado baseado nos documentos abaixo relacionados:

Tipo Documento	Arquivo	Postagem	Autor	Situação
Informações Básicas do Projeto	PB_INFORMAÇÕES_BÁSICAS_DO_PROJETO_2012770.pdf	27/10/2022 19:49:27		Aceito
Outros	respostacep.docx	27/10/2022 19:48:12	VICTOR HIDEKI NAGATANI	Aceito
Cronograma	Cronograma.docx	03/10/2022 11:02:16	VICTOR HIDEKI NAGATANI	Aceito
Folha de Rosto	Folhaa.pdf	03/10/2022 10:29:26	VICTOR HIDEKI NAGATANI	Aceito
Projeto Detalhado / Brochura Investigador	Projeto.docx	20/09/2022 16:00:31	VICTOR HIDEKI NAGATANI	Aceito
TCLE / Termos de Assentimento / Justificativa de Ausência	tcle.docx	20/09/2022 15:57:20	VICTOR HIDEKI NAGATANI	Aceito

Situação do Parecer:
Aprovado

Necessita Apreciação da CONEP:
Não

MOGI DAS CRUZES, 01 de Novembro de 2022

Assinado por:

Marcia Aparecida Silva Bissaco
(Coordenador(a))

Endereço: Av. Dr. Cândido Xavier de Almeida Souza, 200, Prédio 2, Sala 21-21 - Centro Cívico
Bairro: Prédio II, sala 2121(UMC) **CEP:** 08.780-911
UF: SP **Município:** MOGI DAS CRUZES
Telefone: (11)4798-7085 **Fax:** (11)4798-7085 **E-mail:** cep@umc.br
