# Supplementary figures and images for: Farmers’ Perceptions of the Agricultural, Economic, and Health Impacts of Fire Ants in the Brazilian Atlantic Forest
Source: Insects. 2026 Jul 4;17(7):698. doi: 10.3390/insects17070698 (PMC13411350; doi:10.3390/insects17070698)

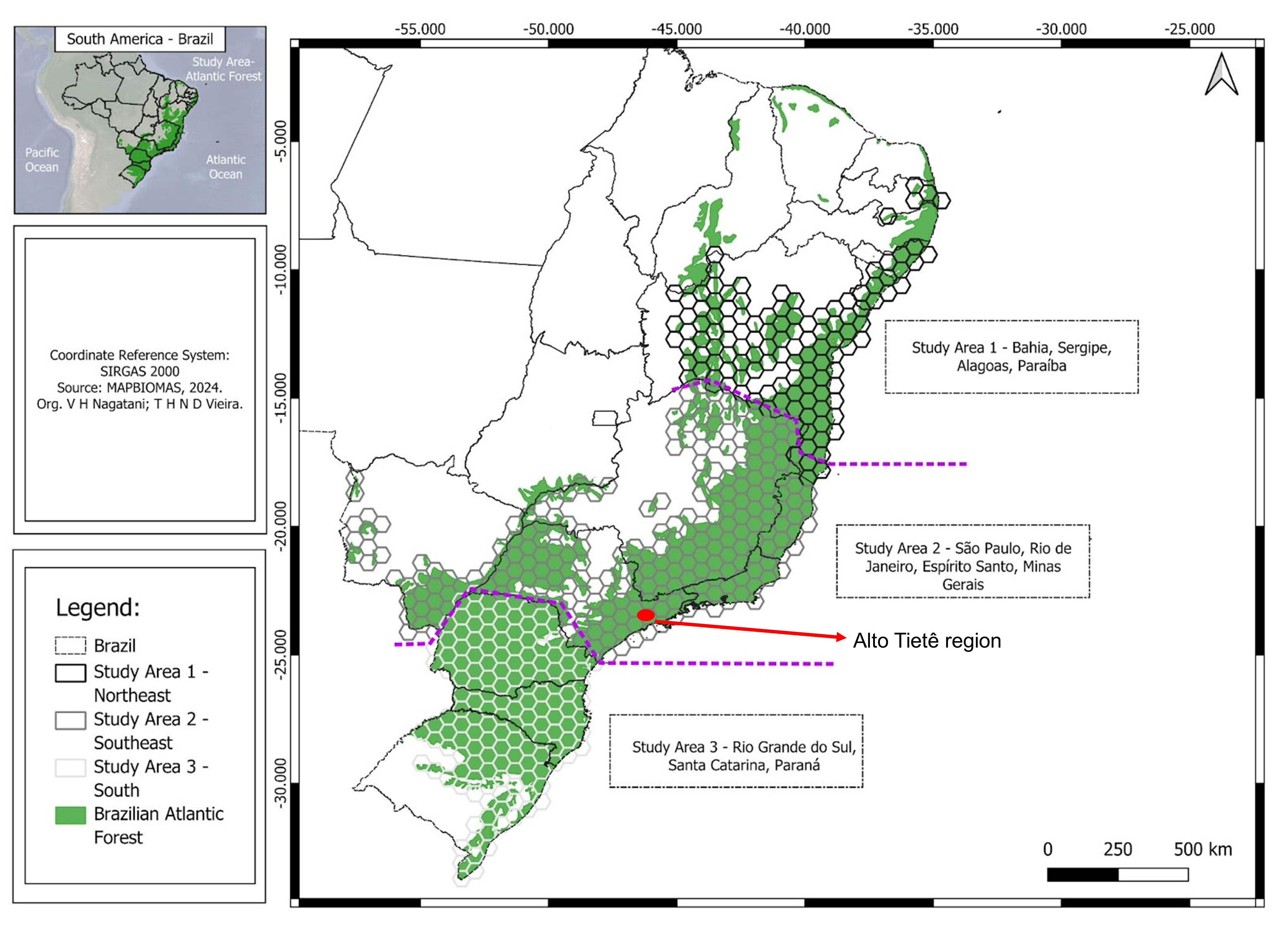

Supplement: Supplementary file 1 [file insects-17-00698-s001.zip › Supplementary Material S1.png]
